# A Convention of Medical Editors

**Published:** 1857-04

**Authors:** 


### *A Convention of Medical Editors.*

The *Southern Journal of the Medical and Physical Sciences* proposes that a convention of the editors of the American medical press should be held during the forthcoming sitting of the American Medical Association at Nashville, "to deliberate upon all subjects pertaining to the support and progress of medical periodical literature." It is a well-known fact, that medical journals in this country do not, as a rule, receive that support from the profession to which they are entitled. A large number of subscribers take their journals regularly without paying for them, or without paying promptly; some from inadvertence, but many, we fear, deliberately. One of the objects of the proposed convention is to institute a reform in this respect, and enable the conductors of the periodical press, not only to be indemnified from loss, but by a reasonable pecuniary return for the expenditure of time and talent to improve the quality of our medical periodical literature, and thus indirectly to elevate the standard of the profession. The effect of good medical journals upon the progress of medicine can hardly be over estimated. As the editor of the *Southern Journal* justly remarks, without them the profession would be "an army without banners, or a ship without sails." It is only by means of a constant interchange of new ideas, the publication of new discoveries, the promotion of friendly feelings throughout the scientific world, that science can advance with those rapid strides which render the present age so remarkable.

It may seem a very easy thing to obtain from subscribers to medical periodicals the small amount which is annually due from them. Experience has shown that in many instances this is not the case, and we suppose that every journal has a certain number, some a large number, of names on its lists, who are not ashamed to receive the periodical without ever paying for it, besides others whose payment is withheld so long, or obtained with such difficulty, as to make it no adequate compensation for the expense incurred by the editor or proprietor. We are therefore glad to see the suggestion of the *Southern Journal*,, and we hope it will be carried into effect. If the majority of the editorial corps will agree to adopt the cash system, and refuse to supply subscribers who are in arrears, until all accounts are settled, we are confident that there will be no reason to regret the reform. The only subscribers lost will be those who do not pay, and hence the result will be an actual gain to the proprietor; while if all journals will unite in this plan, the delinquents will not be able, as is sometimes the case, to supply themselves by running in debt for another periodical. We think a convention of editors might also have a favorable effect upon our medical periodical literature, by deliberating upon the best means of improving the character of our journals, by obtaining a larger amount of valuble original matter, both on the science of medicine and on the ethics of our profession. It is surprising to see how small a space is generally devoted to original articles, and how inferior many of these are in quality. Many of our medical periodicals are chiefly composed of extracts from other journals, and we could name one, at least, whose short existence was almost sustained by matter transferred from our own pages. The subject of *advertisements* is one which should come under the notice of the convention; and considering the profit yielded by this department, and the importance of its effects upon the character of our profession, it is surprising that so little should have been said on this point.

We have briefly referred to a few of the topics which would naturally come before the editorial convention. There are several others which would also form appropriate subjects for discussion, should the proposition of the editor of the *Southern Journal* meet with favor, which we sincerely hope will be the case.—*Boston Med. & Surg. Journal.*

